# Transcriptome Analysis of Sugarcane Young Leaves and Protoplasts after Enzymatic Digestion

**DOI:** 10.3390/life12081210

**Published:** 2022-08-09

**Authors:** Demei Zhang, Rui Wang, Shijian Han, Zhigang Li, Jiming Xiao, Yangrui Li, Lingqiang Wang, Suli Li

**Affiliations:** 1 Guangxi Key Laboratory of Sugarcane Biology, College of Agriculture, Guangxi University, 100 Daxue Rd., Nanning 530004, China; 2 Guangxi Colleges and Universities Key Laboratory of Crop Cultivation and Tillage, Guangxi University, 100 Daxue Rd., Nanning 530004, China; 3 Guangxi Academy of Agricultural Sciences, 174 Daxue Rd., Nanning 530007, China

**Keywords:** sugarcane, protoplast, transcriptome, enzymatic digestion

## Abstract

Sugarcane somatic cell hybridization can break through the barrier of genetic incompatibility between distantly related species in traditional breeding. However, the molecular mechanisms of sugarcane protoplast regeneration and the conditions for protoplast preparation remain largely unknown. In this study, young sugarcane (ROC22) leaves were enzymatically digested, and the viability of protoplasts reached more than 90% after enzymatic digestion (Enzymatic combination: 2% cellulase + 0.5% pectinase + 0.1% dissociative enzyme + 0.3% hemicellulase, pH = 5.8). Transcriptome sequencing was performed on young sugarcane leaves and protoplasts after enzymatic digestion to analyze the differences in gene expression in somatic cells before and after enzymatic digestion. A total of 117,411 unigenes and 43,460 differentially expressed genes were obtained, of which 21,123 were up-regulated and 22,337 down-regulated. The GO terms for the 43,460 differentially expressed genes (DEGs) were classified into three main categories: biological process, cellular component and molecular function, which related to developmental process, growth, cell proliferation, transcription regulator activity, signal transducer activity, antioxidant activity, oxidative stress, kinase activity, cell cycle, cell differentiation, plant hormone signal transduction, and so on. After enzymatic digestion of young sugarcane leaves, the expressions of *GAUT*, *CESA*, *PSK*, *CyclinB*, *CyclinA*, *CyclinD3* and *cdc2* genes associated with plant regeneration were significantly down-regulated to 65%, 47%, 2%, 18.60%, 21.32%, 52% and 45% of young leaves, respectively. After enzymatic digestion, *Aux*/*IAA* expression was up-regulated compared with young leaves, and *Aux*/*IAA* expression was 3.53 times higher than that of young leaves. Compared with young leaves, these key genes were significantly changed after enzymatic digestion. These results indicate that the process of somatic enzymatic digestion process may affect the regeneration of heterozygous cells to a certain extent.

## 1. Introduction

Somatic cell hybridization is a breeding method in which crop plants are fused by somatic cells to obtain regenerated plants. It is characterized by the ability to transfer genes from one species to another, which provides an effective means of breaking the barriers to hybridization before and after fertilization of distantly related and unrelated species in traditional breeding. [1]. Somatic cell fusion (protoplast fusion) technology has been used for crop improvement such as breeding resistant lines [2,3] and combinations of cytoplasmic genes [4], especially in Solanaceae and Brassicaceae. However, only a few hybrid cereals, a particularly important group of plants, have been obtained by this technique due to lack of available approach for efficient plant regeneration from protoplasts. Regarding the success of somatic hybrid breeding in the grass family, it is currently limited to interspecific hybrids in wheat [5,6], intergeneric hybrids between wheat and maize [7], intergeneric hybrids between rice and mangrove [8] and interspecific hybrids in rice [9]. Up to now, somatic cell fusion regeneration in plants still encounters some bottlenecks. 

Plant somatic cell fusion through cell wall separation (enzymolysis) and passivation includes a series of processes, and every step is likely to damage cells [10,11]. The influence of chemical factors on the regeneration ability of heterozygous cells is often the result of environmental conditions and gene interaction. Physiological, biochemical and morphological changes occur throughout the whole process of somatic cell fusion, which is closely related to the gene expression [12]. Protoplast isolation is a stressed process for plants, for example; it enhances the expression of genes involved in regulating heat shock response in magnolias [13]. And the content of RNA, DNA and total nucleic acid in fresh sugarcane young leaves protoplasts obtained after enzymatic digestion was reduced [14]. Sugarcane protoplast culture has been studied for long, but mainly focusing on the isolation and fusion of sugarcane protoplasts, optimization of culture conditions and physiological and biochemical changes [15]. The process of sugarcane protoplast isolation has certain effects on physiology and biochemistry and has significant effects on sugarcane genotypes. Limited by genotype, only a few successful cases of regenerated plants have been reported; however, the experimental repeatability was very low [16]. Sugarcane genotypes are highly specific, and the protoplasts obtained by isolating different genotypes of sugarcane varieties have different regeneration abilities [17]. However, which gene regulation causes the difference in regeneration ability? Which gene regulation is involved in the physiological and biochemical changes? How do hormones induce heterozygous cell regeneration and how do regulatory molecular networks regulate cell division and differentiation to further root and differentiate into seedlings? It is necessary to study the molecular mechanism of sugarcane somatic cell fusion using transcriptome sequencing technology to analyze the differentially expressed genes (DEGs) in the process of somatic cell fusion through functional enrichment analysis. There is no report about the gene function annotation for sugarcane somatic cell heterozygote regeneration.

This study used sugarcane young leaves as materials for transcriptome sequencing to screen the differentially expressed genes related to plant regeneration, to study the molecular mechanism of hybrid cell regeneration through analyzing the key gene expression in the process of somatic cell enzymolysis and chimera cell regeneration, which may provide a reference for further studying the causes of heterozygous cell regeneration difficulties and the molecular regulation mechanism of heterozygous cell regeneration in the future, to improve the success rate of regeneration and achieve accurate regulation of heterozygous cell regeneration in sugarcane.

## 2. Result

### 2.1. Detection of Protoplast Activity in Sugarcane

**Figure 1:**
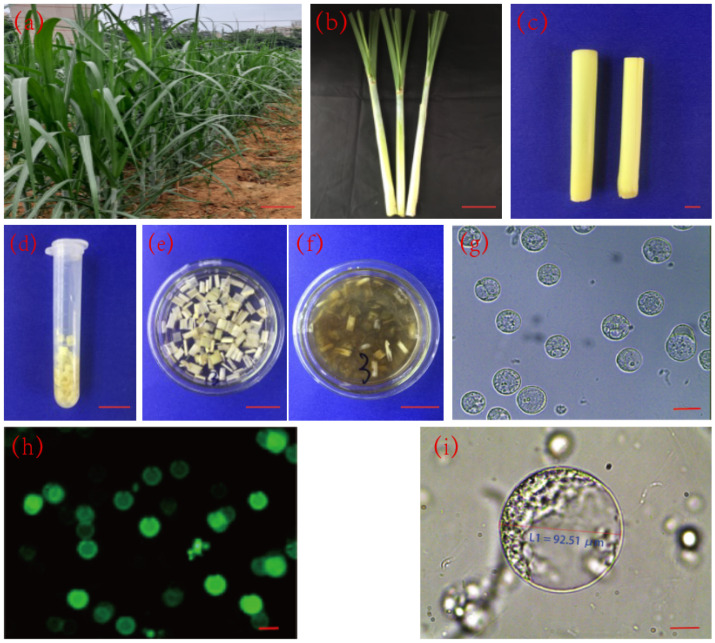
Sugarcane protoplast preparation. (**a**) sugarcane plant at the early elongation stage, bar = 30 cm; (**b**) the outer 2–3 layers of the leaf sheath were peeled off, bar = 7.5 cm; (**c**) the light and yellow central part of young leaves; (**d**) the young leaf cells 1–5 cm above the growing point were cut into sections of about 1 mm in thickness; (**e**) 5 mL of 13% CPW solution was added; (**f**) 5 mL of enzymatic digestion solution was added and enzymatic digestion was carried out for 4 h at room temperature (28 °C); (**g**) after enzymatic digestion sugarcane protoplasts were obtained; (**h**) the dark field of FDA staining of the enzymatically digested sugarcane protoplasts; (**i**) Enlarged protoplasts after enzymatic digestion; Note: (**c**–**f**) bar = 1.5 cm; (**g**–**i**) bar = 20 μm.

The top part of the stem with sheaths of sugarcane at early elongation stage (Figure 1a) was selected to take young leaves samples for enzymatic digestion. The outer two or three sheaths were peeled off (Figure 1b) and the central parts of young leaves (Figure 1c) were taken and sterilized. The cut central young leaves one to five cm above the growing point were cut into sections of about one mm in thickness, put into a test tube and culture dish (Figure 1d,e) and enzymatic digestion solution was added (Figure 1f). The produced protoplast suspension was collected and purified (Figure 1g–i). The Fluorescein diacetate (FDA) detection results showed that the viability of the enzymatically digested sugarcane protoplasts activity was above 90% (Figure 1h).

### 2.2. Quantitative Analysis of Differentially Expressed Genes by RNA-Seq

**Figure 2:**
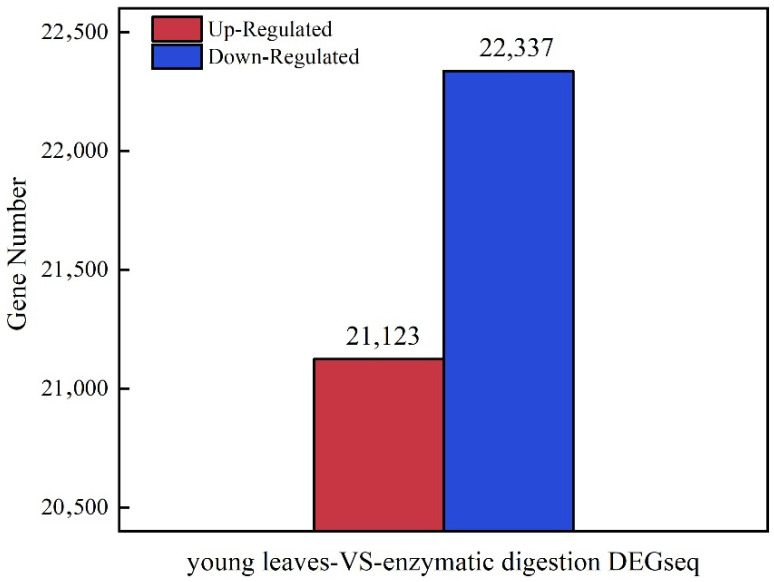
The numbers of differently expressed genes (DEGs) before and after protoplast isolation.

We identified 43,460 differentially expressed genes (DEGs) in protoplasts VS young leaves after enzymatic digestion with the set threshold at 0.05. A total of 43,460 genes were identified as DEGs by RNA-seq. Of them, 21,123 were up-regulated while 22,337 were down-regulated (Figure 2).

Trinity was used to assemble clean reads, and the assembly quality was shown in Table 1. In total, we obtained 117,411 unigenes. Raw data (raw reads) of fastq format were firstly processed through in-house perl scripts. In this step, clean data (clean reads) were obtained by removing reads containing adapter, reads containing ploy-N and low-quality reads from raw data. All the downstream analyses were based on clean data with high quality. The original raw data from Illumina HiSeq^TM^ were transformed into sequenced reads by base calling. Raw data are recorded in a FASTQ file, which contains sequence information (reads) and corresponding sequencing quality information.

**Figure 3:**
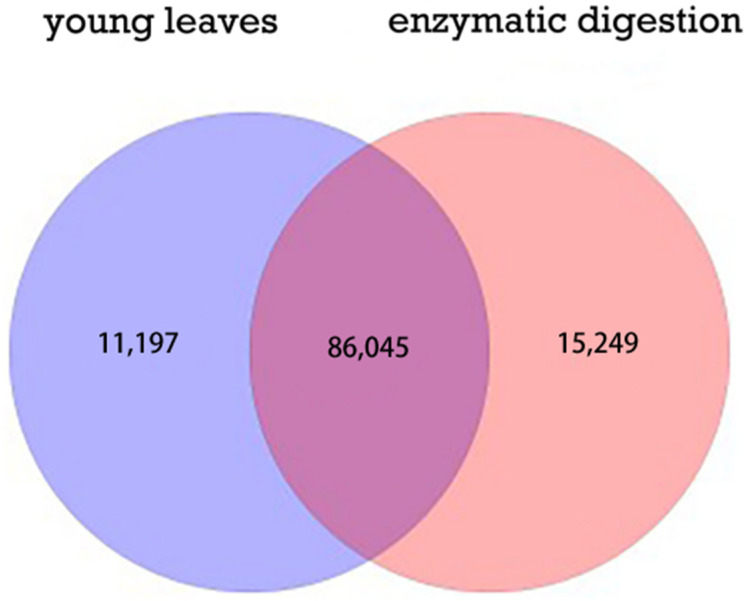
Venn diagram of differently expressed genes.

Quantitative analysis of differential genes expression in young sugarcane leaves before and after enzymatic digestion showed that 86,045 DEGs (76.49%) were common between these two assemblies, 11,197 DEGs (9.95%) unique to those in the sample before enzymatic digestion, and 15,248 DEGs (13.56%) unique to those in the sample after enzymatic digestion (Figure 3).

### 2.3. Validation of RNA-Seq Data by Quantitative RT-PCR

**Figure 4:**
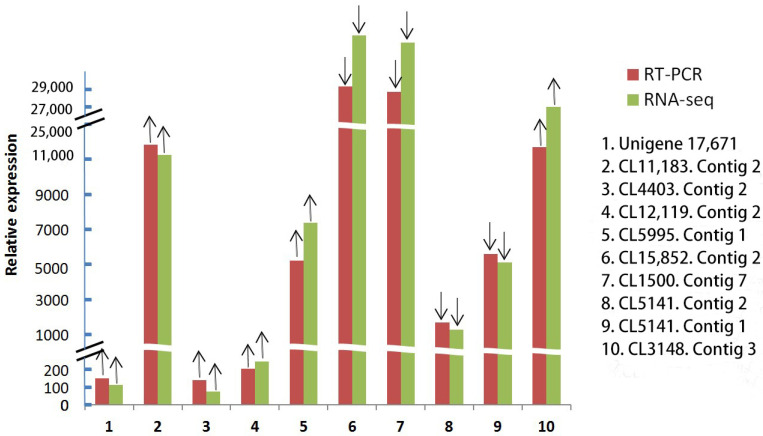
Results of relative expression of selected 10 differentially expressed genes by qRT-PCR. ↑ indicates up-regulated gene expression; ↓ indicates down-regulation of gene expression.

To validate the RNA-seq results, ten genes were randomly selected for relative expression analyses by quantitative PCR (qRT-PCR). In all cases, the up-and-down trend of the fluorescence quantitative results of the selected DEGs was consistent with the RNA-seq results, indicating that the results were reliable. However, there were slight differences in the expression levels of these genes due to the differences in the sensitivity and accuracy of fluorescence quantification and RNA-seq (Figure 4). 

### 2.4. Cluster Analysis of Differential Gene Expression Levels

**Figure 5:**
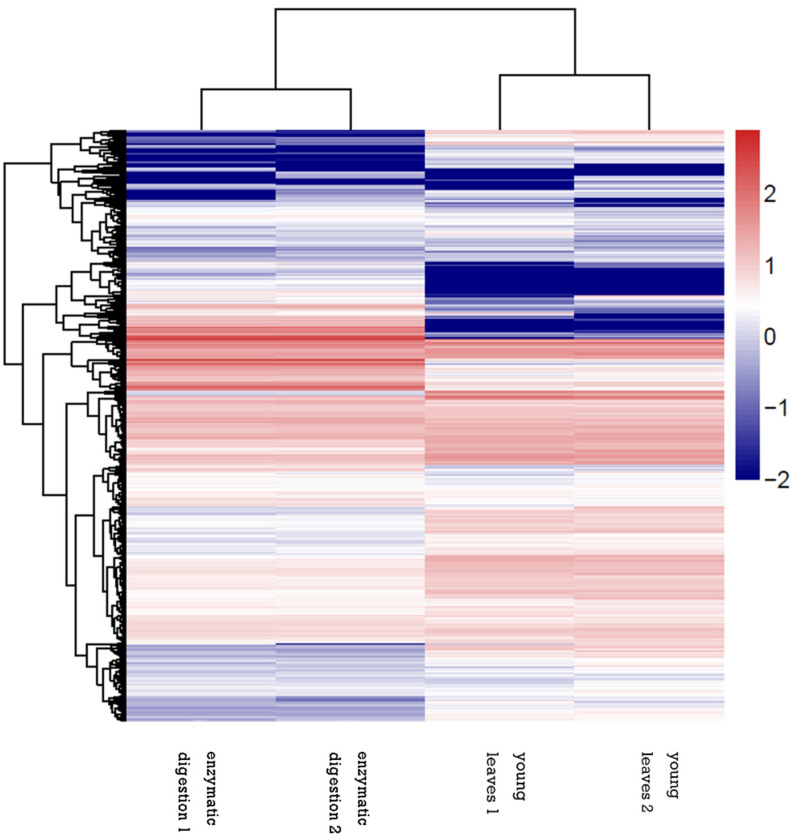
Heatmap representing the distribution of differentially expressed genes (DEGs) in different samples. Red means up regulation, and blue means down regulation, and the deeper the color is, the higher relative expression.

After identification of the DEGs in the samples before and after enzymatic digestion of young sugarcane leaves, the dynamic changes of gene expression patterns were recovered by using a gathering cut-off value of fold change (FC) >2 and an adjusted *p*-value (FDR) of <0.05. By similar expression kinetics, 370 DEGs were depicted by a heatmap analysis to show significant clusters of DEGs according to tissue specificity and time treatment. Similar expression profiles were observed for replicated samples, and specific clusters were denoted for the samples before and after enzymatic digestion of young sugarcane leaves, which reflected considerable difference between the treatments of young leaves and enzymatic digestion(Figure 5).

### 2.5. GO Function Analysis of DEGs

**Figure 6:**
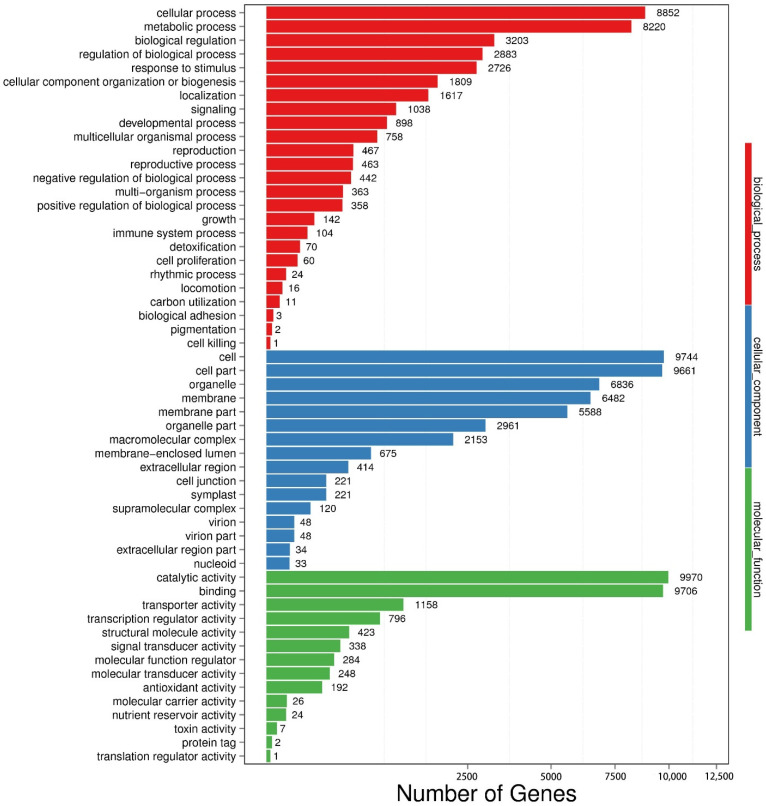
Go enrichment analysis of young leaves VS enzymatic digestion. The X-axis represents the number of differentially expressed genes (DEGs), and the Y-axis represents the GO functional classification.

Figure 6 presents the results of GO analysis for the DEGs between the two sets of transcripts between the treatments of young leaves VS enzymatic digestion identified through BLAST2 GO analysis. The GO terms of 43,460 DEGs were classified into three main categories: biological process (BP, 11,605 transcripts), cellular component (CC, 13,446 transcripts) and molecular function (MF, 15,992 transcripts). Among the biological process, most of the transcripts were assigned to cellular process (8852 transcripts), metabolic process (8220 transcripts) and biological regulation (3203 transcripts). In the cellular component category, the highest proportions of the transcripts were involved in cell and cell part (19,405 transcripts) and organelle (6836 transcripts). In molecular function, catalytic activity was prominent (9970 transcripts), followed by binding (9706 transcripts). 

**Figure 7:**
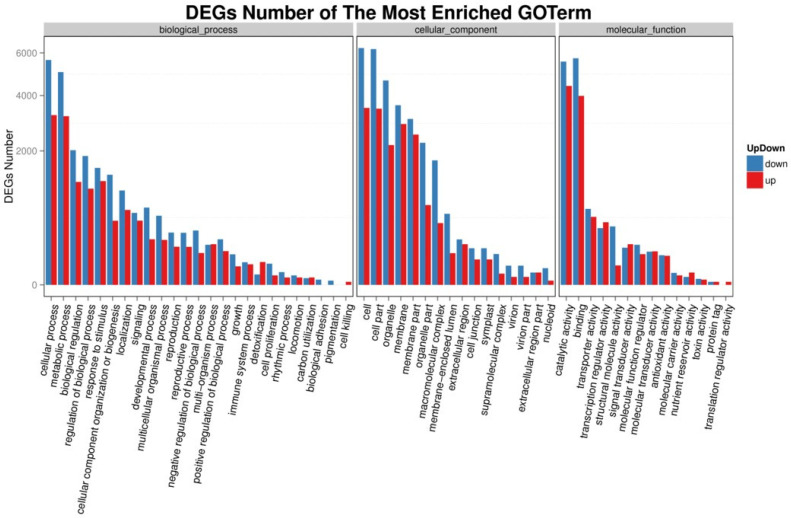
GO annotation histogram for up and down regulation of the differentially expressed genes (DEGs) beteen young leaves VS enzymatic digestion. The X-axis represents the GO functional classification, and the Y-axis represents the number of up and down regulated DEGs in the corresponding GO Term.

Gene Ontology (GO) analysis was employed to identify the important functional groups of the total DEGs between the treatments of young leaves VS enzymatic digestion and visualize the transcript expression. The GO-term annotation suggested that a large proportion of the DEGs were attributed to developmental process, reproductive process, growth, cell proliferation, transcription regulator activity, signal transducer activity, antioxidant activity, oxidative stress, kinase activity, cell cycle, cell differentiation and so on. The DEGs related to these processes are closely related to the cell wall regeneration, cell division and differentiation of freshly isolated protoplasts during culture (Figure 7). 

### 2.6. KEGG Enrichment Analysis of DEGs

**Figure 8:**
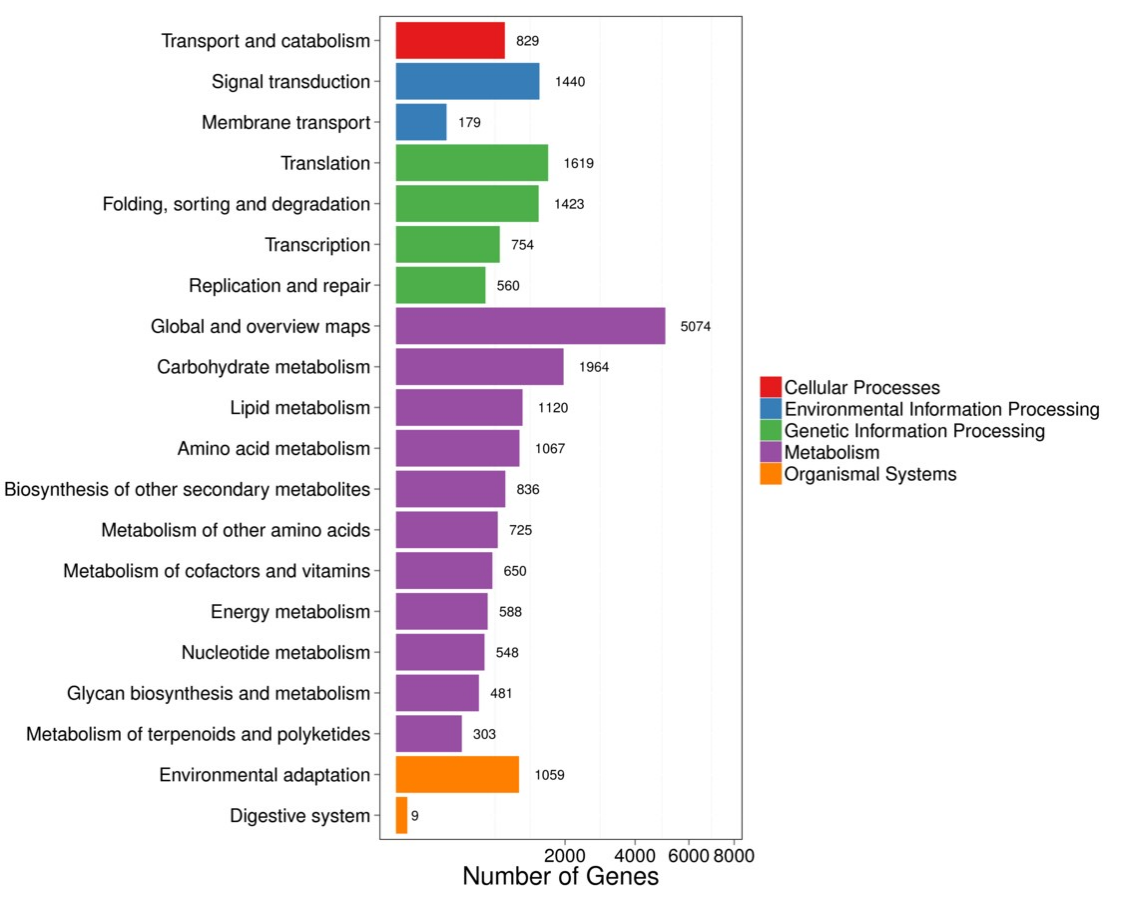
Classification of KEGG metabolic pathways of young leaves VS enzymatic digestion. The X-axis represents the corresponding number of differentially expressed genes (DEGs) and the Y-axis represents the KEGG functional classification.

**Figure 9:**
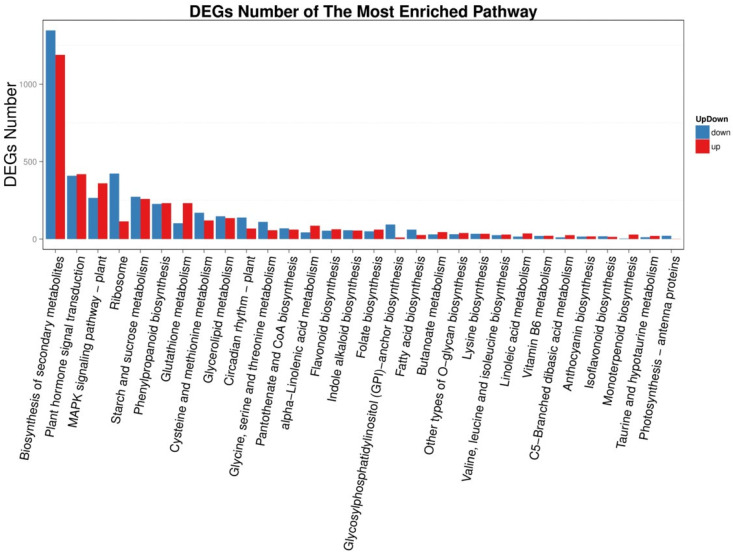
Up and down regulation of KEGG metabolic pathways of young leaves VS enzymatic digestion. The X axis represents the pathway, and the Y axis represents the number of up and down regulated differentially expressed genes (DEGs) for the corresponding pathway.

KEGG metabolic pathway analysis showed that the 21,228 DEGs between young leaves and enzymatic digestion samples were enriched in 20 pathways (Figure 8), and the parameters of KEGG pathway enrichment analysis were listed (Table 2). Genes regulating important pathways such as phytohormone signaling, glutathione metabolism, MAPK signaling pathway-vegetation, glycine, serine and threonine metabolism and monoterpene biosynthesis were heavily up- and down-regulated after enzymatic digestion (Figure 9).

### 2.7. Analysis of Related DEGs in Young Sugarcane Leaves before and after Enzymatic Digestion

Of the 3856 DEGs unique in the young sugarcane leaves before and after enzymatic digestion of young leaves, 2266 transcripts were down-regulated and 1590 up-regulated, respectively (Table 3). The most predominant were “protein kinase activity” containing 802 DEGs, the terms development, signal transduction, cell wall, defense response had 558, 523, 412, 346 DEGs assigned, respectively, while 1 to 189 DEGs were annotated as other GO terms. Amongst them, plant hormone signal transduction, the genes related to cell death in oxidative stress response were down-regulated only. In MAPK signaling pathway, the DEGs related to cell death were up-regulated only. The biological processes cell differentiation, cell proliferation, cell wall, and oxidative stress are important, which may affect plant regeneration. The DEGs associated with these biological processes were found to be heavily down-regulated after enzymatic digestion. These categories represented the common transcriptional functions differentially expressed that were detected in both plant cells before and after enzymatic digestion of young leaves in this study.

### 2.8. Differential Expression of Regeneration Key Genes in Young Leaves before and after Enzymolysis

**Figure 10:**
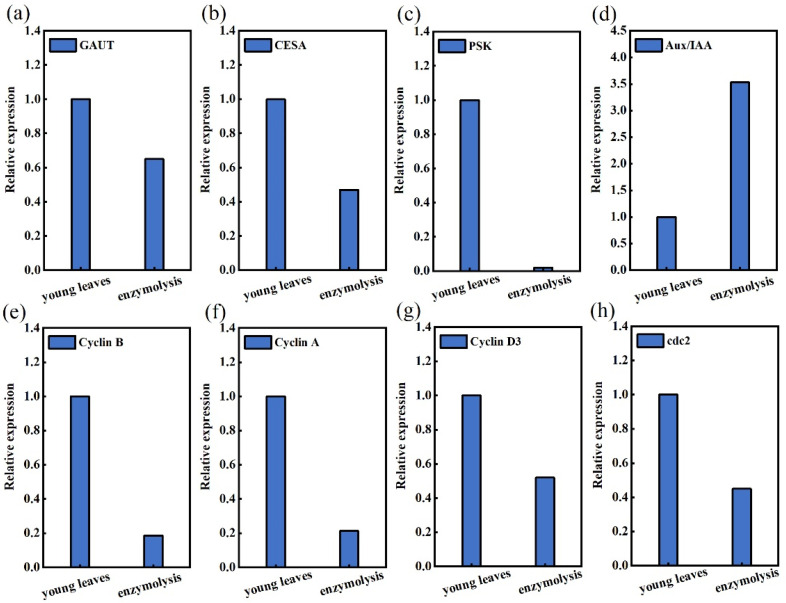
Expression of *GAUT*, *CESA*, *PSK*, *Aux*/*IAA*, *Cycliin B*, *Cyclin A*, *Cyclin D3* and *cdc2* genes before and after enzymolysis of young leaves. (**a**) The expression of GAUT after enzymatic digestion was only 65% of that of young leaves; (**b**) The expression of CESA after enzymatic digestion was only 47% of that of young leaves; (**c**) The expression of PSK after enzymatic digestion was only 2% of that of young leaves; (**d**) The expression of Aux/IAA was up-regulated by 3.53-fold in young leaves after enzymatic digestion; (**e**) The expression of CyclinB after enzymatic digestion was only 18.60% of that of young leaves; (**f**) The expression of CyclinA after enzymatic digestion was only 21.32% of that of young leaves; (**g**) The expression of Cyclin D3 after enzymatic digestion was only 52% of that of young leaves; (**h**) The expression of cdc2 after enzymatic digestion was only 45% of that of young leaves.

Amongst the common DEGs, remarkable ones included those encoding several enzyme proteins associated with cell wall regeneration, cell cycle, cell proliferation and plant hormone signal transduction. The DEGs involved in the young leaves before and after enzymolysis were further investigated. They were divided into four categories: cell wall regeneration genes (Galacturonosyltransferase gene, *GAUT*; cellulose synthase gene, *CESA*), cell cycle-related genes (*CyclinA*, *cyclinB*, *cyclin D3*, *cdc2*), cell proliferation-related genes (phytosulfokine gene, *PSK*) and plant hormone signal transduction-related genes (*Aux*/*IAA*). After enzymolysis of the young sugarcane leaves, the expression levels of the following genes decreased gradually, and *GAUT* expression was only 65% of the original. The expression of *CESA* was only 47% of the original. Compared with the young leaves before enzymolysis, the expression level of *PSK* in protoplasts was very low, which was only 2% of that in the young leaves. After enzymolysis, *Aux*/*IAA* expression was up-regulated compared with that in the young leaves, and *Aux*/*IAA* expression was 3.53 times higher than that in the young leaves. *Cyclin B* expression was significantly down-regulated after enzymolysis, accounting for 18.60% of that in the young leaves only. The expression level of *Cyclin A* was significantly down-regulated, which was only 21.32% of that in the young leaves. The expression level of *Cyclin D3* in protoplasts treated by enzymolysis was significantly lower than that in the young leaves (only 52%). The expression of *CDC2* was similar to *Cyclin D3*, which was only 45% of that in the young leaves (Figure 10). 

## 3. Discussion

### 3.1. Enzymatic Hydrolysis Had a Significant Effect on Gene Expression in Sugarcane Protoplast

The successful isolation of protoplasts depended on breaking down the cell wall and releasing the intact protoplasts [18]. Currently, most protoplasts are produced by enzymatic hydrolysis. The efficiency of this method is influenced by several factors such as the combination of enzyme mixtures, osmotic pressure stabilizer and enzymolysis time [19]. The main components of plant cell walls are pectin, cellulose, hemicellulose and a small amount of proteins. Different enzymes have different effects on cell wall degradation, and removal of cell wall during enzymatic digestion also affected the regeneration of cell wall and plant after somatic cell fusion [20]. Scholars found that the ratio of liquid enzyme cellulase, pectinase, and hemicellulase in the mixed enzyme solution is the key factor determining the quality and yield of protoplasts [21]. The cellulase-pectinase enzymolysis liquid has a great influence on the quality and yield of alfalfa leaf protoplasts [22]. The maceronases and chemicals regulating osmotic pressure affect the physiological and biochemical metabolism of protoplasm, thus affecting the regeneration of heterozygous cells. Meanwhile, the physiological and biochemical level of protoplasts is regulated by molecular level, and the difference in gene expression affects the regeneration ability of plants [23]. The extraction of sugarcane protoplast RNA has the problems of easy degradation, easy browning and low yield, which may be due to the improper conditions in the process of enzymatic digestion which will lead to cell rupture and further increase of phenolic, polysaccharide and secondary metabolites, thus affecting the extraction effect of RNA [24]. The higher the protoplast viability, the higher the RNA yield, because when the protoplast viability was lower than 70%, electrophoresis results showed that RNA was largely degraded into small fragments, and RNA integrity was poor, which could not be used in subsequent molecular experiments. When the protoplast activity was higher than 70%, the purity and integrity of extracted RNA were higher [24]. In our work, the optimal enzymatic conditions for young sugarcane leaves protoplasts were obtained by optimizing the enzyme solution combination, enzymatic concentration, mannitol concentration of CPW and enzymatic time (Enzymatic combination: 2% cellulase + 0.5% pectinase + 0.1% dissociative enzyme + 0.3% hemicellulase, 9%CPW solution at room temperature after 4 h of treatment, pH = 5.8), isolation reached 5 × 10^6^ protoplasts/g FW. The viable protoplasts stained with FDA were measured by fluorescence microscopy under bright light and fluorescence light, and protoplasts were isolated with viability above 90%. Overall, we have established a highly productive and active method for isolation of sugarcane leaf pulp protoplasts, which lays the foundation for subsequent transcriptome analysis and provides reliable material for future genomic studies of sugarcane protoplasts.

### 3.2. The Effect of Enzymatic Hydrolysis on Regeneration Related Genes May Lead to Difficulties in Plant Regeneration from Protoplasts

Our study found that DEGs were enriched in 20 pathways, and most related to metabolism, such as glutathione metabolism, glycine, serine, threonine metabolism, and monoterpene metabolism were significantly down-regulated, and the key genes related to heterozygous cell regeneration (cell wall synthesis, cell cycle, regulation of cell proliferation) were found among them. The expression levels of DEGs in hormone signal transduction pathway were significantly different between sugarcane young leaves and protoplasts after enzymatic digestion, and these key DEGs were significantly down-regulated after enzymatic digestion, suggesting that enzymatic digestion may have a certain degree of adverse influence on heterozygotic cell regeneration. Xu et al. (2021) identified these genes that are not only required for but also greatly promote protoplast regeneration, in particular, callus formation [12].

**Figure 11:**
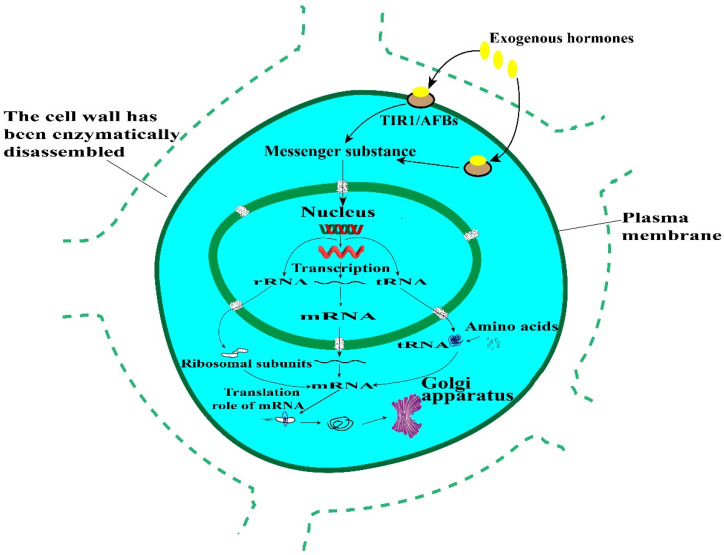
Hypothetical model of exogenous hormone induced cell growth.

Cellulose, hemicellulose and pectin are the three main components of plant primary cell walls. Galacturonic acid transferase (Galacturonosyltransferase, GAUT) is the key enzyme catalyzing UDP-galacturonic acid to form homogalacturonan frame in pectin bio-synthesis [25,26]. Cellulose synthase (CesAs) and cellulose synthase-like (Csl) genes encode enzymes that synthesize cellulose and most hemicellulosic polysaccharides [27]. Loss of GAUT function in the synthesis of pectin and xylan in 15 wild-type Arabidopsis species affects plant cell wall production [28]. GAUTs were highly expressed in the secondary wall thickening stage during the initial stage of fiber development of island cotton, and both the initial stage of fiber development and the secondary wall thickening stage were closely related to the formation of plant cell wall. GAUT 13 and GAUT I4 are located in the Golgi apparatus and are involved in plant development processes, such as promoting the synthesis of pectin and xylan in pollen tube walls and nutrient cell walls [29]. In our study, one of the striking findings was that after enzymatic digestion of young leaves of sugarcane, the expressions of the genes encoding proteins GAUTs and CESAs were down-regulated significantly, which will affect the biosynthesis of pectin and cellulose, possibly leading to the difficulty in cell wall regeneration of protoplasts after enzymatic digestion. The regeneration and synthesis of new cell wall is the first and critical step in the regeneration of somatic cell fusion. It has been shown that exogenous hormones can regulate the synthesis of new protoplasmic components and new cell wall substances in protoplasts due to the fact that growth hormone can promote the synthesis of new nucleic acids and proteins, thus inducing cell growth. And the expression of GAUT and CESA genes are closely related to cell wall components, so in the next research work, we can try to culture sugarcane protoplasts with suitable exogenous hormones to increase the expression of GAUT, CESA, and thus promote the synthesis of new cell walls, as shown in Figure 11.

Peptide signaling is an integral part of cell-to-cell communication which helps to relay the information responsible for coordinating cell proliferation and differentiation. Phytosulfokin Receptor (PSKR) is a transmembrane LRR-RLK family protein with a binding site for small signaling peptide, phytosulfokine (PSK). PSK signaling through PSKR promotes normal growth and development and also plays a role in defense responses [30]. Matsubayashi et al. (1997) discovered and extracted PSK from the suspension cell culture medium of rice and found that PSK can promote the rapid proliferation of plant cells. If mesophyll protoplasts were kept in G0/G1 phase with inhibitors, protoplasts could continue cell cycle normally only when auxin, cytokinin and PSK was present in the medium [31]. PSK can stimulate cell proliferation when cell division activity is low, and this process requires the participation of auxin 2,4-D [32]. This study found that, compared with young sugarcane leaves, the expression level of PSK gene changed significantly after enzymatic hydrolysis, from 1 to 0.02, indicating that the significantly reduced expression level of PSK gene significantly affected cell proliferation and development, probably resulting in the difficulty of somatic regeneration of sugarcane after fusion.

Aux/IAA is the initial response gene of growth hormone, which is the hub of the growth hormone signaling process [33]. When growth hormone is at low concentration, Aux/IAA is synthesized in large quantities and the activity of growth hormone response factor (AUXIN RESPONSE FACTOR, ARF) is inhibited; when growth hormone is at higher concentration, the expression of Aux/IAA is degraded by ubiquitination and ARF is activated, and the activated ARF regulates downstream genes controlling the growth hormone signaling pathway [34]. The results of this study showed that the expression of Aux/IAA was up-regulated after enzymatic degradation compared with fresh young leaves, which indicated that the enzymatic digestion of the cells of young sugarcane leaves significantly affected the expression of Aux/IAA, which might have a certain effect on the response of protoplasts to regulate the growth hormone signaling process during heterozygous cell culture.

The cell cycle proteins Cyclin A and Cyclin B form a complex with cell cycle-dependent protein kinase (CDK) to regulate cell cycle progression [35,36]. Cyclin A forms a complex with CDK2 to promote the forward progress of S phase, and Cyclin B combines with CDK1 to form a maturation-promoting factor to regulate mitosis [37,38]. Once the protoplasts have completed the process of regenerating a cell wall, they will immediately re-enter the cell cycle in order to initiate cell division, which in turn allows the cells to proliferate and grow [39,40]. The results of this study showed that the expression of Cyclin A and Cyclin B were down-regulated in young sugarcane leaves protoplasts after enzymatic digestion, which indicated that the enzymatic digestion in the somatic cell fusion of sugarcane significantly affected the expression of CDK2, which might have adverse effect on the response of protoplasts to regulate cell cycle progression during heterozygous cell culture.

The expression of CyclinD3, a cell cycle protein, is highly dependent on cytokinin [41,42]. Cdc2, a cyclin-dependent kinase, controls cell cycle progression, exogenous hormones can also promote cell division by regulating cdc2. The results of this study showed that the expression of CyclinD3 was down-regulated in young sugarcane leaves protoplasts after enzymatic digestion [43,44]. The results of this study showed that the expression of CyclinD3 and cdc2 was down-regulated after the process of enzymatic digestion compared to fresh young sugarcane leaves, which indicated that the treatment in the somatic cell fusion of sugarcane significantly affected the expression of CyclinD3 and cdc2, which might affect the response of protoplasts to regulate exogenous phytohormones during heterozygous cell culture.

## 4. Material and Method

### 4.1. Plant Material Preparation

The sugarcane variety ROC22 was used, and the young leaves were sampled at early elongation stage. The samples were quick-frozen in liquid nitrogen and sorted at −80 °C for further use.

The separation of protoplast from young sugarcane leaves was done based on the method proposed by Song (2018) [45] with slight modification. The robust tail sheaths of sugarcane at the initial stage of elongation were selected as the enzymic hydrolysis materials of young leaves (Figure 1a). The outer 2–3 layers of the leaf sheaths were peeled off first and then sterilized with 75% alcohol for 30 s. After being washed with sterile water 3 times, the outer layer and the leaf sheaths at both ends were removed to expose the light-yellow central leaves. The young leaves 1–5 cm above the growing point were cut into slices with a thickness of about 1 mm. 0.5 g of young leaves were collected and put into 5 mL of CPW solution (containing 13% mannitol, pH 5.8). After plasmolysis for 0.5–1 h, the CPW (containing 13% mannitol) solution was removed and 5 mL of enzymic hydrolysis solution was added to perform the enzymic hydrolysis at room temperature for 4 h. Then, the protoplast suspension was collected through 100 and 200 mesh cell sieves, and the protoplasts were purified by gradient centrifugation. After enzymatic hydrolysis, the density of protoplasts was adjusted to 1 × 10^6^/mL, 0.5 mL were taken and shaken well, then frozen in liquid nitrogen and stored at −80 °C. Two replicates were set.

### 4.2. Preparation of RNA-Seq Libraries

Total RNA was extracted from the materials before and after enzymatic hydrolysis of young sugarcane leaves. Agarose gel electrophoresis was performed on the extracted RNA, and Agilent 2100 Bioanalyzer (Agilent RNA 6000 Nano Kit, Manufacturer: Agilent Technologies Inc.; Source: Santa Clara, CA, USA) was used to determine the total RNA concentration, RIN value, 28S/18S and fragment size of the samples. After the RNA was qualified, the total RNA was processed by enriching the mRNA with polyA tail by Oligod magnetic beads. The RNA obtained was segmented by interrupting buffer, and the random N6 primers were reversely transcribed, and then the two strands of cDNA were synthesized to form double-stranded DNA. The synthetic double-stranded DNA ends were smoothed and phosphorylated at the 5′ end to form a sticky end protruding a “A” at the 3′ end, and then connected with a bubbling-like connector protruding a “T” at the 3′ end. The ligands were amplified by PCR with specific primers. The PCR products were thermally denatured into single strands, and the single strand DNA was cycled with a bridge primer to obtain a single strand circular DNA library, which was then sequenced on Illumina platform.

### 4.3. Quality Control of Sequencing Data and Identification of Differentially Expressed Genes

The full-length transcriptome sequencing was carried out, and a total of 26.48 Gb of data were obtained from RNA-seq using BGISEQ-500 platform. First, the sequencing joints were pruned, and then reads with unknown base N content greater than 5% and low-quality reads were removed (defined as low-quality reads with the proportion of base number less than 10 in total number of the reads greater than 20%). After clean reads were obtained in the following analysis, trinity was used for de novo assembly of clean reads, and tgicl was used for clustering de-redundancy of transcripts to obtain unigene. According to the assembly results, unigene was used, the clean reads of each sample were compared to unigene by Bowtie2 software (Version Number: Bowtie2; Creator: Ben Langmead & Steven L Salzberg; Source: College Park, MD, USA), and then the gene expression level of each sample was calculated by RSEM. According to the method of Wang et al. (2010) [46], DEGseq detection was performed, the difference multiple was more than twice and the Q-value was 0.001, screened as significantly differentially expressed genes.

### 4.4. Functional Annotation for DEGs

To define the function of DEGs, enrichment analyses of Gene Ontology (GO) and Kyoto Encyclopedia of Genes and Genomes (KEGG) pathways were performed using the Phyper function in R software (Version Number: 4.2.0; Creator: the R Core Team; Source: Auckland City, New Zealand), followed by FDR correction for *p*-value. Generally, the GO item at FDR 0.01 and the KEGG pathway at FDR 0.01 were considered to be significantly enriched.

### 4.5. Real-Time Quantitative PCR Analysis of Genes

According to the transcriptome data results, 10 genes were randomly selected from the differentially expressed genes for fluorescence quantitative PCR validation to determine the reliability of the RNA-seq results. Primer 3.0 was used to design the primers, and the primer sequences were shown in Table 4. The specific steps of RNA reverse transcription to cDNA synthesis were PrimeScript^TM^ II Qrt-pcr was performed using TaKaRa’s TB Green^TM^ Premix Ex Taq^TM^ II Kit. RT-PCR was performed according to the TB Green^TM^ Premix Ex Taq^TM^ II kit from TaKaRa, and the amplification system was 20 μL and the amplification procedure was as follows: 95 °C 30 s, 1 cycle; quantitative analysis: 95 °C 5 s, 60 °C 30 s, 40 cycles; melt curve: 95 °C 5 s, 60 °C 1 min, 95 °C, 1 cycle; cooling: 95 °C 30 s, 1 cycle.

### 4.6. Data Statistical Analysis

Excel 2010 was used for data calculation, and a SPSS19.0 statistical software(Version Number: 19.0; Creator: IBM Corporation; Source: Armonk, NY, USA) package was used to conduct one-way analysis of variance and test the significant difference between groups.

## 5. Conclusions

**Figure 12:**
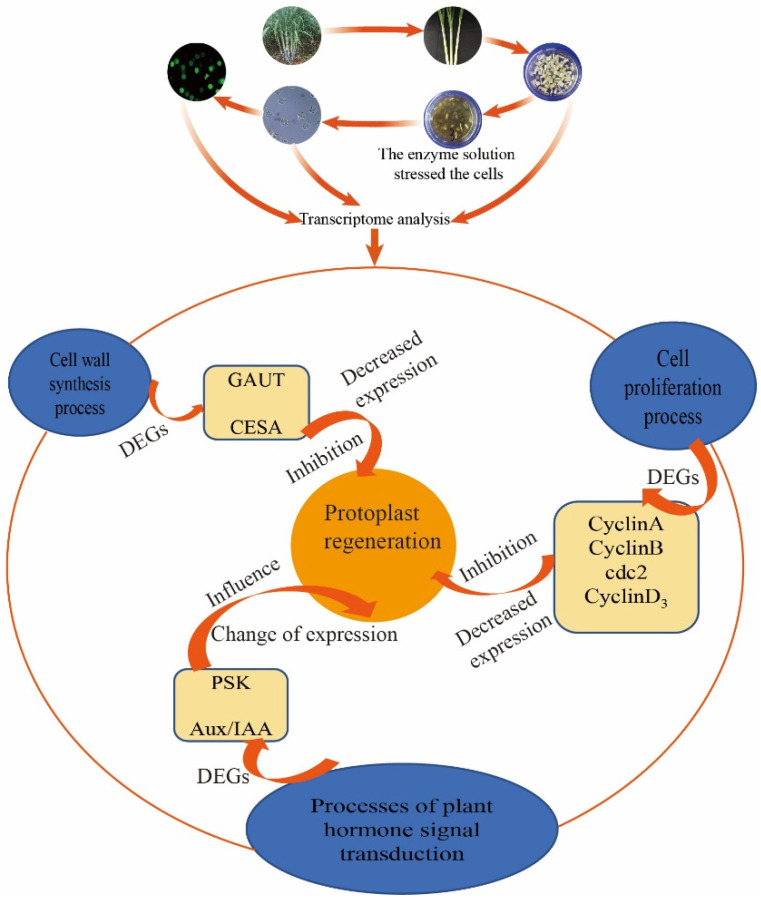
Transcriptome analysis of young sugarcane leaves after enzymatic digestion, affecting protoplast regeneration.

In our study, transcriptome data analysis revealed that the expression of regeneration-related genes such as cell wall synthesis, cell cycle regulation, cell proliferation, hormone signaling pathways, somatic embryogenesis genes, and oxidative stress genes were significantly different after enzymatic digestion of young sugarcane leaves compared to before enzymatic digestion. Key genes such as GAUT, CESA, CyclinA, CyclinB, CyclinD3, cdc2, and PSK were significantly down-regulated after enzymatic digestion and significantly changed after Aux/IAA enzymatic digestion compared to young leaves. These results suggest that the enzymatic digestion process may affect the genes of cell regeneration to some extent, which is the molecular basis of the difficulty of sugarcane protoplast regeneration. The impact hypothesis was modeled as follows (Figure 12):

## Figures and Tables

**Table 1:** The statistics of unigene assembly results.

Sample	Total Number	Total Length	Mean Length	N50	N70	N90	GC (%)
enzymatic digestion 1	78,382	73,051,813	931	1560	949	369	49.07
enzymatic digestion 2	77,229	72,426,174	937	1569	960	372	49.22
young leaves 1	67,147	65,903,089	981	1611	1028	397	50.23
young leaves 2	68,813	66,219,582	962	1598	1002	388	50.14
Total unigenes	117,411	1.29 × 10^8^	1102	1800	1186	474	49.35

**Table 2:** KEGG pathway enrichment analysis for young leaves VS enzymatic digestion.

Pathway	Gene Number	Background Gene Number	Rich Factor	Q-Value
Glutathione metabolism	334	540	0.618518519	6.42 × 10^−6^
Pantothenate and CoA biosynthesis	130	197	0.659898477	4.60 × 10^−4^
Photosynthesis—antenna proteins	22	24	0.916666667	9.50 × 10^−4^
Circadian rhythm—plant	207	340	0.608823529	2.08 × 10^−3^
C5-Branched dibasic acid metabolism	36	46	0.782608696	2.61 × 10^−3^
Starch and sucrose metabolism	532	945	0.562962963	3.13 × 10^−3^
Glycosyl phosphatidylinositol (GPI)-anchor biosynthesis	104	161	0.645962733	3.68 × 10^−3^
Ribosome	537	958	0.560542797	3.80 × 10^−3^
Glycerolipid metabolism	282	490	0.575510204	1.26 × 10^−2^
Folate biosynthesis	111	179	0.620111732	1.54 × 10^−2^
Vitamin B6 metabolism	41	59	0.694915254	2.82 × 10^−2^
Phenylpropanoid biosynthesis	459	833	0.551020408	3.84 × 10^−2^
Butanoate metabolism	75	119	0.630252101	3.85 × 10^−2^
Glycine, serine and threonine metabolism	168	290	0.579310345	5.70 × 10^−2^
Monoterpenoid biosynthesis	32	46	0.695652174	5.97 × 10^−2^
Lysine biosynthesis	68	109	0.623853211	6.59 × 10^−2^
Plant hormone signal transduction	828	1551	0.53384913	7.14 × 10^−2^
Cysteine and methionine metabolism	290	522	0.555555556	7.41 × 10^−2^
Alpha-linolenic acid metabolism	129	221	0.583710407	7.41 × 10^−2^
Indole alkaloid biosynthesis	112	192	0.583333333	1.09 × 10^−1^

**Table 3:** GO analysis of differentially expressed genes (DEGs) in young leaves before and after enzymatic digestion.

GO Term	Number of Up-Regulated Genes	Number of Down-Regulated Genes
Cell cycle	36	80
Development	132	426
Cell differentiation	40	104
Cell wall	154	258
Cell proliferation	10	50
Oxidative stress	84	105
MAPK signaling pathway	2	0
Cell death	17	17
Growth	42	102
Cell killing	1	0
Transcription regulator activity	42	71
Antioxidant activity	1	5
Plant hormone signal transduction	0	3
Protein kinase activity	465	337
Signal transduction	215	308
response to external stimulus	72	108
Morphogenesis	21	49
Post-embryonic development	2	7
Defense response	177	169
Response to stress	60	36
Cell death in response to oxidative stress	0	2
Regulation of response to reactive oxygen species	8	20
Osmotic regulation	9	9

**Table 4:** Primers for qRT-PCR.

Gene ID	Primer 5′—3′	
Unigene17671_All	F	CCGAGACCAAAGACATCTTGC
	R	GGGATCAGCTTCGTCATCAC
CL11183.Contig2_All	F	GGGCTACTCGAAGCTGATTG
	R	CGAATCGGACTCTAGGGTTGA
CL4403.Contig2_All	F	CCTACGCCGATTTCTACCAG
	R	GCTTGCCAAAGACTTGCCTC
CL12119.Contig2_All	F	GCACTACCAGCATGGGTTTAG
	R	GCTCCCGTGGCATACTACAA
CL5995.Contig1_All	F	GGCACAGGGCTAGTTTTAGAC
	R	CCACCAGAGTACATTCCACG
CL15852.Contig2_All	F	CAAGAAGGCTGGCAGGTGGAAG
	R	CACGAGCAAGTCCTCTGACAGTTC
CL1500.Contig7_All	F	AAGGATGTGAATGCCGCTGTGG
	R	CGCTGGTGGAGTTGGAGATCATG
CL7155.Contig2_All	F	AGGCGGAATGATAGGTCGAGGTC
	R	CGTCGTAGCGGTCGGAGGAG
CL5141.Contig1_All	F	CCAGTTCTGCCTCAACCACTTCTC
	R	GTGCCTGCCGTCTGCTTCTC
CL3148.Contig3_All	F	CCAGTCGCCATCACCATCATCATC
	R	CTCCTCCTCGCCGCTGTCAG
GADPH	F	AAGGGTGGTGCCAAGAAGG
	R	CAAGGGGAGCAAGGCAGTT

## Data Availability

All the data generated in this study are present in the main manuscript.
